# Assessment of innate immune response modulating impurities in glucagon for injection

**DOI:** 10.1371/journal.pone.0277922

**Published:** 2022-11-21

**Authors:** Qingxia Han, Zhongli Bao, Mary Ziping Luo, Jack Yongfeng Zhang

**Affiliations:** Amphastar Pharmaceuticals, Inc., Rancho Cucamonga, California, United States of America; Brandeis University, UNITED STATES

## Abstract

Glucagon for Injection is a polypeptide hormone medication used to treat patients with severe hypoglycemia or low blood sugar. Only recently, was a generic version of glucagon for injection approved by the FDA. While the generic version was deemed equivalent to its brand-name counterpart, the two glucagon products were produced using different manufacturing processes. The brand-name glucagon is produced via recombinant DNA while the generic glucagon is produced by peptide synthesis. Different manufacturing processes can result in different levels of innate immune response modulating impurities (IIRMIs). This study utilized a cell-based assay method, which allows for detection of a broad spectrum of impurities, to investigate the IIRMI risks of the generic glucagon to make sure it has similar or less immunogenicity risks than the brand-name glucagon product. Three commercial cell lines (RAW-Blue™, HEK-Blue™-hNOD1 and HEK-Blue™-hNOD2) carrying a secreted embryonic alkaline phosphatase reporter construct were used to quantify the level of innate immune responses after being treated with the glucagon drugs. The study results showed that despite differences in manufacturing process, the innate immunogenicity risk in the synthetic (generic) glucagon was at negligible level and comparable to the recombinant (brand-name) glucagon product.

## Introduction

Glucagon for Injection is a polypeptide hormone medication used to treat patients with severe hypoglycemia or low blood sugar [1]. The brand-name version, which was approved over 20 years ago, was developed by Eli Lilly & Company and is supplied as a lyophilized powder to be reconstituted with diluent for immediate use. Only recently (December, 2020), was a generic version of glucagon for injection (1 mg/vial packaged in an emergency kit) approved by the FDA. While the generic was deemed equivalent to its brand-name counterpart, the two glucagon products were produced using different manufacturing processes [1,2]. The brand-name glucagon is produced via recombinant DNA while the generic glucagon is produced by peptide synthesis. Therefore, there might be some process-related impurities in the generic (synthetic) glucagon product, which can potentially induce stronger or wider unwanted innate immune responses in patients.

The innate (or inborn) immune system is the first line of immune system that provides immediate but non-specific responses to foreign substances [3,4]. This is achievable through various cell surface or intracellular pattern recognition receptors (PRRs), which are capable of recognizing molecules found in pathogens called pathogen-associated molecular patterns (PAMPs). Recognition of PAMP by PRR initiates a downstream signaling cascade that leads to the activation of nuclear transcription factor NF-kB, which in turn results in the induction of cytokines and chemokines that promote inflammation and enhance adaptive immune responses [4,5]. Studies have shown that even trace levels of process and product related impurities could act as PAMPs. These impurities, known as innate immune response modulating impurities (IIRMIs), can greatly compromise the safety and efficacy of the drug product by triggering unwanted innate and adaptive immune responses to peptide products, such as producing anti-drug antibodies [6–8]. These anti-drug antibodies may lead to severe and potentially lethal clinical consequences such as loss of therapeutic efficacy and/or the formation of autoimmunity [9–13]. Therefore, it is crucial to investigate the levels of these IIRMIs that may be present in drug products such as glucagon.

While detecting IIRMIs and assessing the risk of immunogenicity is critical, previous testing strategies are often limited to the use of the LAL (limulus amebocyte lysate) test to measure endotoxin, a PCR test to detect host cell DNA, and ELISA based tests for host cell proteins [8,10]. To broaden the spectrum of IIRMIs testing, a well-established cell-based assay method was developed by Haile *et al*., which screens products for the presence of IIRMIs and assesses their immunogenic risk [6]. In their study, Haile *et al*. presented two approaches that allow for the detection of a broader subset of IIRMIs. In the first approach, commercial cell lines transfected with specific PRR receptors are used to detect receptor-specific agonists. This method is sensitive to trace levels of IIRMIs and provide information of the type of IIRMIs, but requires prior knowledge of the possible impurities that might be present in the product. The second approach uses a combination of macrophage cell lines of human and mouse origin to detect a broad range of impurities and therefore is more useful when the impurities are unknown.

In this study, both of the above-mentioned approaches were used to evaluate the innate immunogenic risks of the generic (synthetic) product, along with the brand-name (recombinant) glucagon products. The results demonstrated that the innate immunogenic risk of the generic glucagon product is as low and negligible as the brand-name glucagon product.

## Materials and methods

### Preparation of glucagon lots

Three recently manufactured lots (one-month shelf life) and three aged lots (24-month shelf life) of generic (synthetic) glucagon for injection were provided by Amphastar Pharmaceuticals, Inc. (Rancho Cucamonga, CA). Six lots of recombinant glucagon for injection (Eli Lilly and Company, Indianapolis, IN) were purchased commercially, of which three were more recently released (6–9 month shelf life) and three were near expired (18-month shelf life). All samples were freshly prepared and tested immediately after reconstitution per product’s labeling instructions. In this study, the synthetic glucagon is referred to as AMP-glucagon and the recombinant glucagon is referred to as ELI-glucagon.

### Cell lines selection

Cell lines were selected based on the potentially-presented or known impurities in the generic synthetic glucagon product. The impurities in the synthetic glucagon were identified to be glucagon-like short peptides, which chemically and structurally resemble lipoprotein and peptidoglycan. Therefore, PRR cell lines expressing lipoprotein and peptidoglycan were used in this study.

Three reporter cell lines, RAW-Blue^™^, HEK-Blue^™^-hNOD1 and HEK-Blue^™^-hNOD2 were purchased from InvivoGen (San Diego, CA, USA). RAW-Blue™ cells are derived from the murine RAW 264.7 macrophages with chromosomal integration of a secreted embryonic alkaline phosphatase (SEAP) reporter construct inducible by NF-κB and AP-1. RAW-Blue™ cells express numerous pattern-recognition receptors (PRRs), including toll-like receptors (TLRs), RIG-I-like receptors (RLRs) and C-type lectin receptors (CLRs) and therefore can be used for wide-spectrum of IIRMI screening. However, RAW-Blue™ is not sensitive to NOD-like receptor (NLR) agonists, such as muramyl dipeptide (MDP), which is the minimal bioactive peptidoglycan motif common to all bacteria. HEK-Blue™-hNOD1 and HEK-Blue™-hNOD2 are two cell lines overexpressing human NOD1 and NOD2 receptors respectively. In HEK-Blue™-hNOD1 cells, the SEAP reporter gene is placed under the control of the IFN-β minimal promoter fused to five NF-κB and AP-1 binding sites. In HEK-Blue™-hNOD2 cells, the SEAP reporter gene is placed under the control of the IL-12 p40 minimal promoter fused to five NF-κB and AP-1 binding sites. Stimulation of the three cell lines with respective agonists will lead to the activation of the NF-κB and AP-1 transcription which will in turn result in SEAP production and secretion into the cell culture supernatant. The innate immune response level is positively correlated with the SEAP amount in the cell culture supernatant.

### Cell culture and reagent

Cells were cultured based on the vendor’s respective instructional manual. In brief, cells were grown in complete cell culture medium made of DMEM GlutaMax (Gibco, Cat#10569–010) supplemented with 10% FBS (Gibco, Cat#16140–01) and 1% Pen-Strep (Invitrogen, Cat#1540–122). To maintain stable cell lines and minimize contamination, 200 μg/mL of antibiotic Zeocin (InvivoGen) was added to the growth medium of RAW-Blue™ cells, and 30 μg/mL of antibiotic blasticidin (InvivoGen) along with 100 μg/mL of Zeocin (InvivoGen) were added to the cell culture medium of HEK-Blue^™^-hNOD1 and HEK-Blue^™^-hNOD2 cells.

For routine cell passaging and cell seeding into 96-well plate, cells were first rinsed once with pre-warmed 1x Hank’s Balanced Salt Solution (HBSS) (Gibco Cat#14175–095) after the removal of the cell culture medium. Another 5 mL of fresh 1x HBSS was then added to the cells and incubated at 37°C in a CO2 incubator for about two minutes. The RAW-Blue™ cells were detached by scraping while the HEK-Blue™ cells were detached by pipetting up and down gently in 1x HBSS as suggested in the respective instructional manuals. After a centrifugation at the speed of 200 g for 5 minutes, the HBSS supernatant were poured out and the cells were re-suspended in their corresponding complete cell culture medium. Cell numbers were counted using a hemocytometer. A proper number of cells were then plated in cell culture dishes (approximate 1x 10^5^ cells per dish) or 96-well plates (approximate 2.5 x 10^4^ cells per well) and grew at 37°C in a CO2 cell culture incubator. QUANTI-Blue™ and QB buffer, as well as four kinds of PRR agonists (FSL-1, Pam3CSK4, LPS-B5 and M-TriDAP) were also purchased from InvivoGen. The cell viability testing reagent AlamarBlue HS (cat# A50101) were purchased from ThermoFisher Scientific (Waltham, MA).

### Cell viability assay

A cell viability assay was performed with the widely used AlamarBlue method [14,15]. This method uses an indicator dye resazurin, which changes color in response to cellular metabolic activity and in turn, allows for the quantification of cell viability in a sample.

Since AMP-glucagon is the generic version of ELI-glucagon and has the same dosage regimen, a representative lot of AMP-glucagon (1 mg/mL) was used for the cell viability test. The cell treatment procedure is the same as that used in the innate immunogenicity assessment assay. Firstly, 2-fold serial glucagon dilutions with concentrations ranging from 100% to 6.25% were prepared using the 100% glucagon solution (1 mg/mL in its diluent) as the starting solution and the complete cell culture medium as the diluent. Secondly, cells were seeded in a 96-well cell culture plate with 60 μL of cell suspension per well. Thirdly, an equal volume (60 μL) of the respective concentration of glucagon solution was individually added to each well and mixed with the cells. After staying in the biosafety hood for 15–30 minutes to allow an even cell deposition and distribution in the wells, the cell culture plates containing cells incubated in various concentration (50% to 3.125%) of glucagon solutions were transferred to the CO2 cell culture incubator and grew at 37°C for 24 hours.

To test the cell viability after 24-hour drug treatment, the cell culture medium was gently removed and replaced with 120 μL of AlamarBlue (Introgen™ A5010) which was diluted to 10% with cell culture medium. After 4 hours incubation, the optical density (OD) of the cell culture plates was measured at 570/600 nm using a Spectramax microplate reader. “No treatment” control wells were included in each 96-well plate for background OD correction. The 600 nm values were used as the reference wavelength. DMSO, a reagent known to be toxic to cells at higher concentrations, was added to the wells as a positive control at concentrations ranging from 25% to 1.56%. The cell viability experiment of each cell line was conducted three times, and in each experiment, four individual wells were used for each treatment condition, including various concentrations of glucagon or DMSO treatment and the no drug treatment control. Average of the four replicates were calculated for each experiment.

### Innate immunity assessment assay

Cells were treated with 0.25 mg/mL glucagon or a concentration series of a commercial PRR ligand, alone or in combination with glucagon, in 96-well plates at 37°C in a CO2 cell culture incubator. After 24 hours of treatment, 20 μL of cell culture supernatant from each well were collected and mixed with 180 μL of 1x QUANTI-Blue™ solution (InvivoGen, rep-qbs2) in a new 96-well plate. After 4 hours of incubation, SEAP levels were determined colorimetrically by spectrophotometry at a wavelength of 620 nm. Three individual wells were used for each drug treatment condition and the average reading of the triplicates was used to represent the respective run. During each run, AMP-glucagon lots were always tested alongside with ELI-glucagon lots and at least one positive (ligand) and one no-drug-treatment negative control. At least three independent experiments/runs were performed for each cell line and each glucagon lot. Cells were observed using microscopy before and after treatment during each run to ensure that cells were in good growth condition. Cell viability assay was conducted at least once along with the innate immunity assay to also ensure all types of treatment did not impact cell viability.

### Statistical analysis

Individual data points of innate immunity assay represent the mean ± standard deviation of OD values from at least three independent experiment runs. Statistical comparison was assessed by Student’s *t*-test between a drug treatment group and its negative control. One-way ANOVA was used for response comparison among multiple glucagon treatment groups and the “no treatment” control (seven treatment conditions in total). Statistical significance was defined as *p*-value <0.05. If *p*<0.05 by one-way ANOVA test among treatment conditions, two-way factor ANOVA test will be performed to compare between the AMP-Glucagon lots and the ELI-glucagon lots. Microsoft Excel 2016 and GraphPad Prism 8.0 were used for statistical calculation and graph making.

## Results

### Determination of highest concentration of glucagon for cell treatment

Drug treatment may kill cells and render a false negative immune response in a cell-based immunogenicity assessment assay. To determine the highest concentration of glucagon product which can be used for cell treatment in the innate immunogenicity assay, a cell viability assay was first performed with the method described above. Cells were incubated with different concentrations of a representative AMP-glucagon lot for 24 hours and then tested for viability with the widely used AlamarBlue method. The average viability of the “no-drug treatment” control cells was set as 100%. As shown in Table 1, all three cell lines retained over 90% average viability after 24 hours of glucagon treatment at concentrations up to 0.25 mg/mL (25% glucagon in cell culture). When the concentration of glucagon increased to 0.50 mg/mL, the average cell viability was significantly reduced by more than 20% for all three cell lines. Therefore, the highest concentration of glucagon to be used for the innate immunogenicity assessment assay was determined to be 0.25 mg/mL for all three cell lines. As reported [16,17], DMSO treatment of cells showed apparent dose-toxicity correlation for all three cell lines, with less than 20% cell viability left after DMSO treatment at concentrations above 5% as seen in Table 1.

**Table 1:** Summary of the cell viability assay.

Cell lines	AMP-Glucagon Treatment						DMSO Treatment				
	Dose (%)^†^	Exp 1^†^	Exp 2^†^	Exp 3^†^	Mean ± stdev	Dose (%)^†^		Exp 1*^†^	Exp 2^†^	Exp 3^†^	Mean ± stdev
RAW-Blue™	3.125	95.6	103.1	104.4	101.0 ± 4.8	1.56		92.7	104.2	86.4	94.4 ± 9.0
	6.25	100.5	102.8	102.7	102.0 ± 1.3	3.125		58.4	65.0	46.5	56.6 ± 9.4
	12.5	101.2	96.9	98.6	98.9 ± 2.2	6.25		16.4	15.4	13.7	15.2 ± 1.3
	25	105.0	98.5	104.0	102.5 ± 3.5	12.5		13.2	20.2	10.9	14.8 ± 4.8
	**50**	77.3	67.0	94.5	**79.6 ± 13.9**	25		6.2	3.2	3.8	4.4 ± 1.6
HEK-Blue^™^-hNOD1	3.125	99.0	92.5	104.6	98.7 ± 6.1	1.56		64.9	67.1	64.0	65.3 ± 1.6
	6.25	99.3	97.0	101.8	99.4 ± 2.4	3.125		57.4	62.9	50.9	57.1 ± 6.0
	12.5	96.8	95.5	103.4	98.5 ± 4.2	6.25		43.7	43.5	33.0	40.1 ± 6.1
	25	89.6	89.0	97.8	92.1 ± 4.9	12.5		21.5	21.9	15.9	19.8 ± 3.4
	**50**	80.2	27.3	86.4	**64.7 ± 32.5**	25		10.3	5.7	2.6	6.2 ± 3.9
HEK-Blue^™^-hNOD2	3.125	99.3	102.1	102.8	101.4 ± 1.8	1.56		71.2	76.8	81.5	76.5 ± 5.2
	6.25	91.2	96.6	103.6	97.1 ± 6.2	3.125		53.5	62.5	64.1	60.0 ± 5.7
	12.5	91.5	101.2	103.0	98.5 ± 6.2	6.25		43.2	39.4	39.2	40.6 ± 2.3
	25	89.0	99.7	100.7	96.5 ± 12.5	12.5		17.6	18.4	15.8	17.3 ± 1.4
	**50**	84.8	32.7	88.2	**68.6 ± 31.1**	25		6.1	3.8	3.8	4.6 ± 1.3

^†^ Drug percentage in cell culture, made by dilution of the 100% AMP-glucagon (1 mg/mL with its diluent) with complete cell culture medium.

* Values in columns are cell viability percentage relative to that of the “no drug treatment” control (normalized to 100%).

### Innate immunogenicity assessment with the RAW-Blue™ cell line

Proper cell line selection is critical for the cell-based innate immunogenicity assessment assay. Detection of IIRMI impurities in cell-based assays require cells that are sensitive to the presence of IIRMI in the tested product and can elicit a quantifiable response [18]. Since generic glucagon products are chemically synthesized and impurities detected through sensitive HPLC-MS are all peptide-like substances, cell lines with the capability to detect peptide-like ligand stimulation were considered. The RAW-Blue™ cell line (SEAP reporter construct inducible by NF-κB promoter, which will be activated after PRR binding with IIRMIs) is known to express multiple PRRs and has been shown to be sensitive to several peptide-like PAMPs such as diacylated lipoproteins (the ligand to TLR-2 and 6 receptors) and triacylated lipoproteins from bacteria (TLR-2 and 1 receptors) [6]. Therefore, this cell line was used for potential innate IIRMI screening in glucagon products first. In this wide-spectrum innate immunogenicity detection platform, the optical density values obtained are positively correlated to the SEAP reporter level released in the cell culture supernatant, which reflects the innate immune response intensity in the cell culture after drug treatment.

To ensure that the RAW-Blue™ cells used in this study have the expected sensitivity and specificity as reported, three different Toll-like receptor (TLR) ligands (i.e. FSL-1, recognized by PRR combination TLR2+TLR6; Pam3CSK4, recognized by PRR combination TLR1 +TLR2; and LPS-B5, recognized by TLR4) and an NOD1/NOD2 ligand (i.e. M-TriDAP) were used for cell stimulation. Concentration series of the ligands were based on previous literature [6] and vendor’s recommendations. As shown in Table 2, FSL-1, Pam3CSK4 and LPS-B5 induced significantly higher responses compared to the no treatment control at concentrations as low as 0.01 ng/mL, 1 ng/mL and 1 ng/mL, respectively. At these concentrations, the three TLR ligands had average OD values of 0.218, 0.264 and 0.746, respectively, which were distinctly higher than the no treatment groups (0.092, 0.116 and 0.132, respectively). It is noted that the average response of RAW-Blue^TM^ cells to 0.1 ng/mL LPS-B5 was 0.246, which was nearly two-fold of that of the negative control (0.132). However, the difference was not statistically significant with a *p*-value of 0.24 (*p*>0.05). The higher than expected of this *p*-value may be due to the small number of replicates (n = 3) and large variation of individual responses among the triplicates.

**Table 2:** Positive control testing of RAW-Blue™ cell line sensitivity.

Positive Control Ligand	Dose ng/mL	Cell A RAW-Blue™			
		n =	Ave. OD_260_	S.D.	*P*-value
**FSL-1** (synthetic diacylated peptide TLR2/6 ligand)	0	4	0.092	0.010	-
	0.01	4	0.218	0.045	0.0015
	0.1	4	0.419	0.086	<0.001
	1	4	0.774	0.227	0.0010
	10	4	0.875	0.204	<0.001
**Pam3CSK4** **(**synthetic triacylated peptide (TLR2/1 ligand))	0	5	0.116	0.066	-
	0.1	5	0.151	0.079	0.4774
	1	5	0.264	0.124	0.0465
	10	5	0.468	0.147	0.0012
	100	5	0.770	0.239	<0.001
	1000	5	0.963	0.375	0.0011
**LPS-B5** (Endotoxin, TLR4 ligand)	0	3	0.132	0.079	-
	0.1	3	0.246	0.121	0.2400
	1	3	0.746	0.271	0.0196
	10	3	1.264	0.562	0.0259
	100	3	1.352	0.461	0.0107
	1000	3	1.408	0.516	0.0133
**M-TriDAP** (Peptidoglycan NOD1/NOD2 ligand)	0	5	0.116	0.064	-
	100	5	0.119	0.059	0.9355
	1000	5	0.118	0.049	0.9520
	10,000	5	0.146	0.080	0.5309

Highlighted data in yellow indicate the lowest concentration of the ligand which elicited significant higher innate immune response when compared to the negative control (medium only) by Student’s t-test with *p-*value <0.05.

For the NOD1/NOD2 ligand (M-TriDAP), the responses were similar to the negative control at all concentrations tested with *p*-values > 0.05. These results were consistent with the previous report [6] which showed that RAW-Blue™ is highly sensitive and specific in detecting TLR-stimulating impurities, but not sensitive to NOD1 and NOD2 ligands.

**Fig 1:**
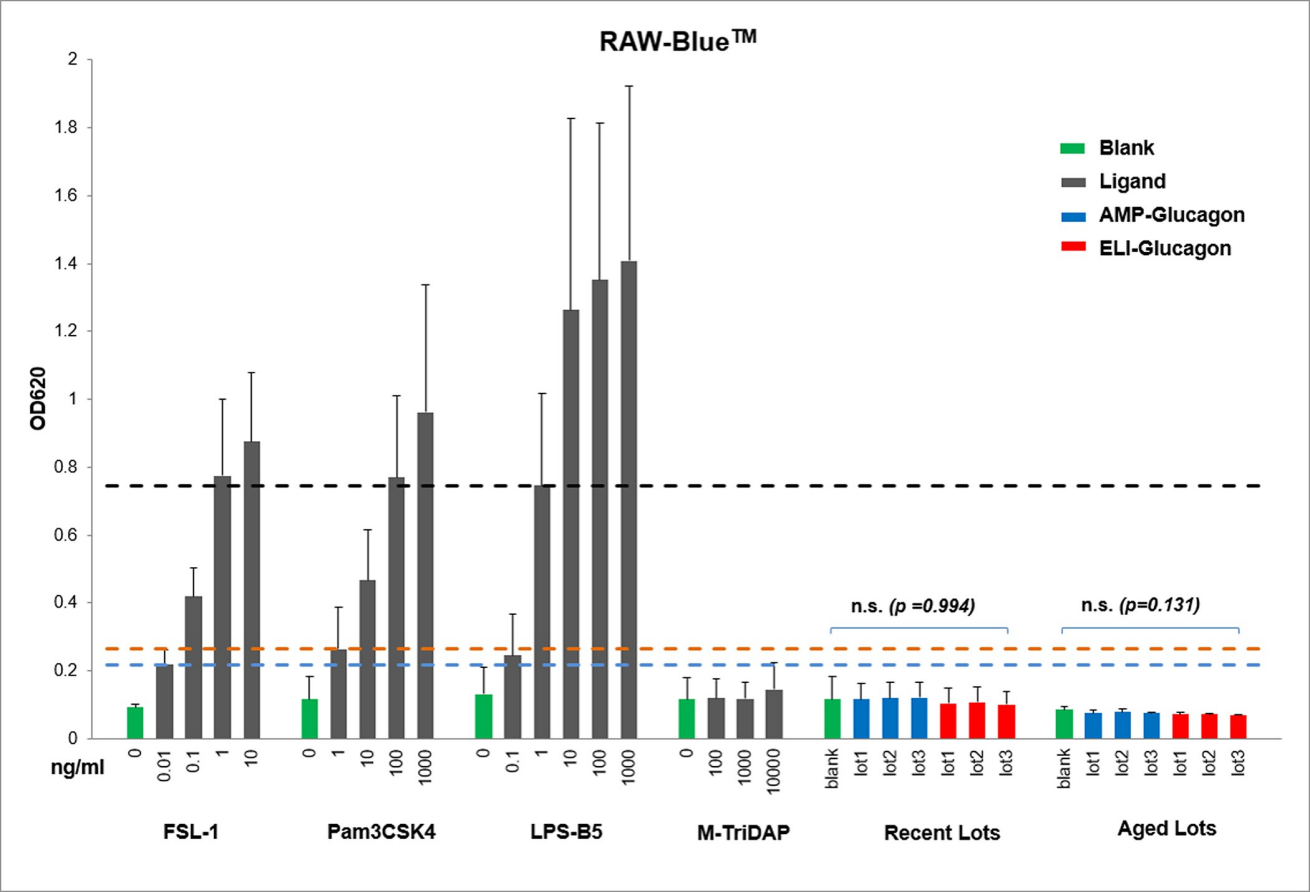
Comparison of innate immune response of AMP-Glucagon and ELI-Glucagon in RAW-Blue™ cells. 2.5 x 10^4^ RAW-Blue™ cells were cultured for 24 hours with increasing concentration of ligands, and three lots of recent and aged AMP-glucagon and ELI-glucagon products. Transcription Factor NF-κB activation was measured from the supernatant using QUANTI-Blue^TM^ method. Each bar represents mean ± stdev of 3–5 repeats. “n.s.” means not significant by one-way ANOVA test. Dash lines represent the LLOD of the ligands.

For innate immunity assessment of glucagon products, AMP-glucagon and ELI-glucagon were tested in cell culture at the maximal concentration of 0.25 mg/mL according to the cell viability assay previously discussed. As demonstrated in Table 3 and Fig 1, the innate immune response levels elicited by ELI-glucagon and AMP-glucagon were comparable, with ELI-glucagon being slightly lower. For the new lots, AMP-glucagon had an average immune response levels of 0.116–0.121 while the ELI-glucagon was 0.101–0.108. A similar pattern was seen in the aged lots where AMP-glucagon had an average response levels of 0.075–0.079 while ELI-glucagon was 0.070–0.073. The responses triggered by both products were considered to be non-significant and were comparable to the no-treatment negative control groups (one-way ANOVA test, *p*>0.05). Further, it is worthwhile to note that the response levels of all glucagon lots are below the lower limit of detection (LLOD) response levels of the positive control ligands as shown in Fig 1. The low levels of transcription factor NF-kB activation by the glucagon products in RAW-Blue™ cell indicate that glucagon products, synthetic or recombinant, had negligible risk of innate immunogenicity that can be detected by RAW-Blue™ cells.

**Table 3:** Innate immunity assay of glucagon products with RAW-Blue^TM^.

Tested Samples			Dose ng/mL	Cell A RAW-Blue™			
				n =	Ave. OD_260_	S.D.	*P*-value
**Recent Manufacturing lots**	Blank	**no treatment** (medium)	0	4	0.117	0.066	0.9943**
	AMP-Glucagon	lot #1, GV002J9 (1 Mo.*)	250000	4	0.116	0.047	
		lot #2, GV001J9 (1 Mo.)	250000	4	0.120	0.048	
		lot #3, GV000J9 (1 Mo.)	250000	4	0.121	0.047	
	ELI-Glucagon	lot #1, D065360A (6 Mo.)	250000	4	0.104	0.045	
		lot #2, D065359D (7 Mo.)	250000	4	0.108	0.045	
		lot #3, D065359C (9 Mo.)	250000	4	0.101	0.037	
**Aged lots**	Blank	**no treatment** (medium)	0	3	0.086	0.008	0.1313**
	AMP-Glucagon	lot #1, 102017B (24 Mo.)	250000	3	0.075	0.010	
		lot #2, 102017A (24 Mo.)	250000	3	0.079	0.010	
		lot #3, 102017 (24Mo.)	250000	3	0.075	0.005	
	ELI-Glucagon	lot #1, C886898C (18 Mo.)	250000	3	0.073	0.005	
		lot #2, C875312A (18 Mo.)	250000	3	0.072	0.001	
		lot #3, C886898C (18 Mo.)	250000	3	0.070	0.003	

*Mo. = month.

**p-values by one-way ANOVA test.

### Innate immunity assessment with HEK-Blue^™^ cell lines

Since RAW-Blue™ cells are not sensitive to NLR ligands, the study further assessed the NOD1/NOD2-stimulating impurities in glucagon products utilizing cell lines HEK-Blue^™^-hNOD1 and HEK-Blue^™^-hNOD2. Experiments were carried out in the same manner as the RAW-Blue^™^ cell line. Three ligands, M-TriDAP, FSL-1, and Pam3CSK4 were used to test the sensitivity and specificity of the two cell lines.

As shown in Table 4, the M-TriDAP which is a NOD1 and NOD2 ligand, induced a significant response at the lowest tested concentration in HEK-Blue™-hNOD1 and hNOD2 cell lines (*p* < 0.001 by Student’s t-test compared to the negative control group). At the LLOD concentration, the M-TriDAP ligand had OD values of 0.322 and 0.735 in the HEK-Blue™-hNOD1 and hNOD2 cell lines respectively, compared to the no treatment control (0.074 and 0.094). In contrast, the FSL-1 and Pam3CSK4 TLR ligands, even at their highest concentrations, did not induce any significant response in the two cell lines and was comparable to the negative control (*p* > 0.05). The results confirmed that HEK-Blue™-hNOD1 and hNOD2 cell lines are sensitive and specific.

**Table 4:** Innate immunity assay with HEK-Blue™-hNOD1 and hNOD2.

Cell Lines Tested Samples			Dose ng/mL	Cell B HEK-Blue™- hNOD1				Cell C HEK-Blue™—hNOD2			
				n =	Ave.	S.D.	*P*-value	n =	Ave.	S.D.	*P*-value
**Positive controls (PC)**	**PC1 (FSL-1),** synthetic diacylated peptide TLR2/6 ligand		0	4	0.073	0.002	-	4	0.096	0.023	-
			0.01	4	0.073	0.002	0.92	4	0.095	0.027	0.98
			0.1	4	0.074	0.002	0.75	4	0.091	0.020	0.74
			1	4	0.073	0.002	0.79	4	0.092	0.027	0.84
			10	4	0.073	0.002	0.81	4	0.087	0.016	0.55
	**PC2 (Pam3CSK4)** synthetic triacylated peptide (TLR2/1 ligand)		0	3	0.074	0.000	-	3	0.096	0.019	-
			0.1	3	0.074	0.002	0.96	3	0.098	0.020	0.91
			1	3	0.072	0.008	0.68	3	0.105	0.028	0.67
			10	3	0.075	0.003	0.66	3	0.108	0.027	0.56
			100	3	0.074	0.001	0.60	3	0.119	0.032	0.35
			1000	3	0.073	0.005	0.57	3	0.138	0.031	0.12
	**PC4 (M-TriDAP)** Peptidoglycan NOD1/NOD2 ligand		0	5	0.074	0.002	-	-	-	-	-
			1000	5	0.322	0.022	<0.001	-	-	-	-
			10,000	5	0.852	0.148	<0.001	-	-	-	-
			0		-	-	-	5	0.094	0.021	-
			100		-	-	-	5	0.735	0.176	<0.001
			1000		-	-	-	5	1.593	0.321	<0.001
			10,000		-	-	-	5	1.338	0.304	<0.001
**Recent Manufacturing lots**	Blank	**no treatment** (medium)	0	4	0.073	0.002		4	0.090	0.009	
	AMP-Glucagon	lot #1, GV002J9 (1 Mo.*)	250000	4	0.112	0.023	0.74**	4	0.095	0.013	<0.001**
		lot #2, GV001J9 (1 Mo.)	250000	4	0.088	0.004		4	0.080	0.007	
		lot #3, GV000J9 (1 Mo.)	250000	4	0.087	0.005		4	0.081	0.009	
	ELI-Glucagon	lot #1, D065360A (6 Mo.)	250000	4	0.095	0.008		4	0.162	0.034	
		lot #2, D065359D (7 Mo.)	250000	4	0.092	0.005		4	0.133	0.037	
		lot #3, D065359C (9 Mo.)	250000	4	0.094	0.008		4	0.165	0.060	
**Aged lots**	Blank	**no treatment** (medium)	0	3	0.072	0.000		3	0.102	0.030	
	AMP-Glucagon	lot #1, 102017B (24 Mo.)	250000	3	0.120	0.057	0.58**	3	0.157	0.070	<0.001**
		lot #2, 102017A (24 Mo.)	250000	3	0.109	0.033		3	0.128	0.043	
		lot #3, 102017 (24Mo.)	250000	3	0.082	0.008		3	0.096	0.028	
	ELI-Glucagon	lot #1, C886898C (18 Mo.)	250000	3	0.099	0.004		3	0.307	0.024	
		lot #2, C875312A (18 Mo.)	250000	3	0.097	0.006		3	0.305	0.085	
		lot #3, C886898C (18 Mo.)	250000	3	0.093	0.005		3	0.284	0.027	

*Mo. = month. Highlighted data in yellow indicate the lowest concentration of the ligand which elicited significant higher innate immune response when compared to the negative control (medium only) by Student’s t-test with *p-*value <0.05.

**p-values by two-factor ANOVA test.

**Fig 2:**
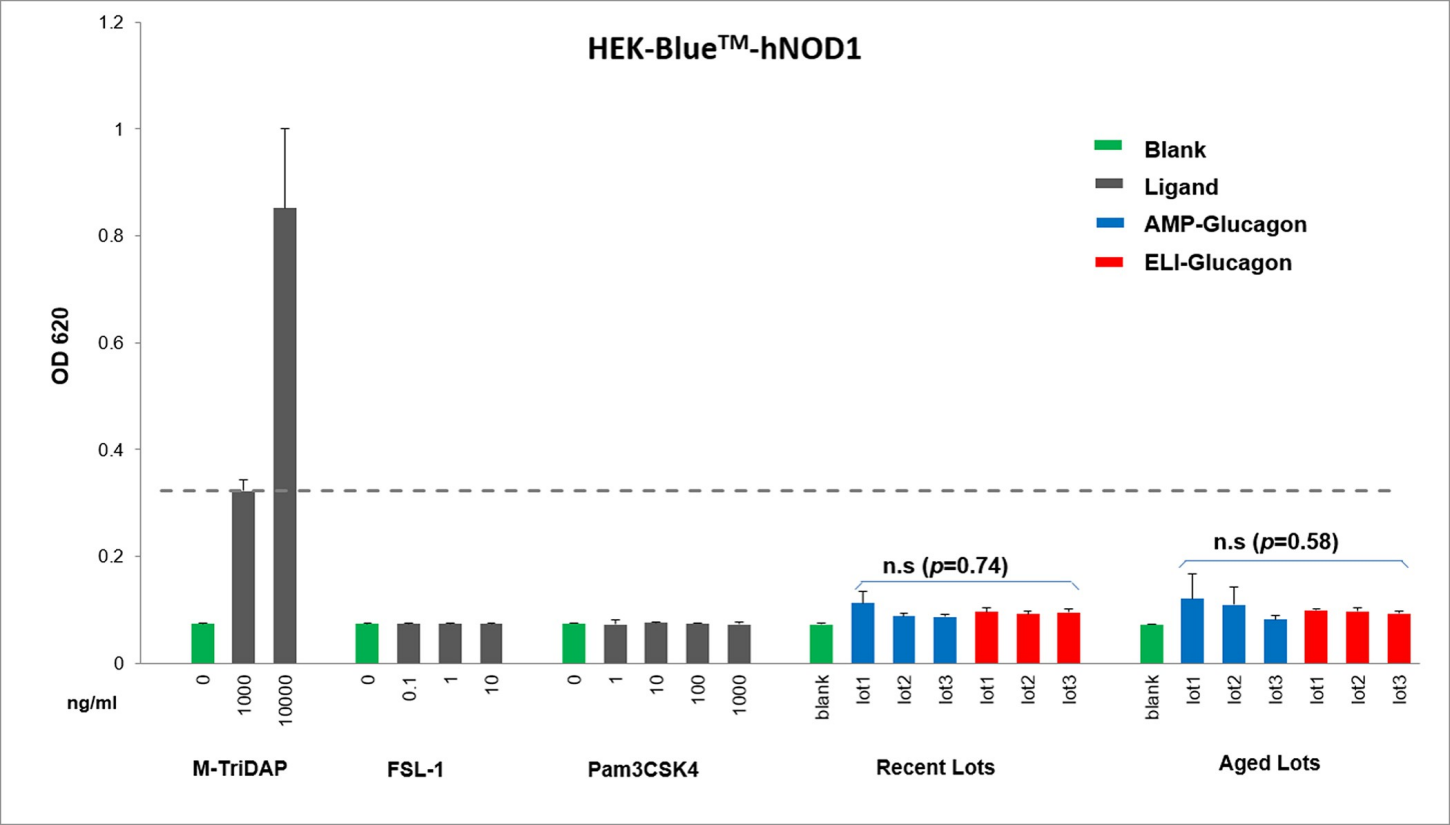
Comparison of innate immune response of AMP-Glucagon and ELI-Glucagon in HEK-Blue™-hNOD1 cells. Each bar represents mean ± stdev of 3–5 times of independent experiments. n.s, no significance by two- way ANOVA test.

**Fig 3:**
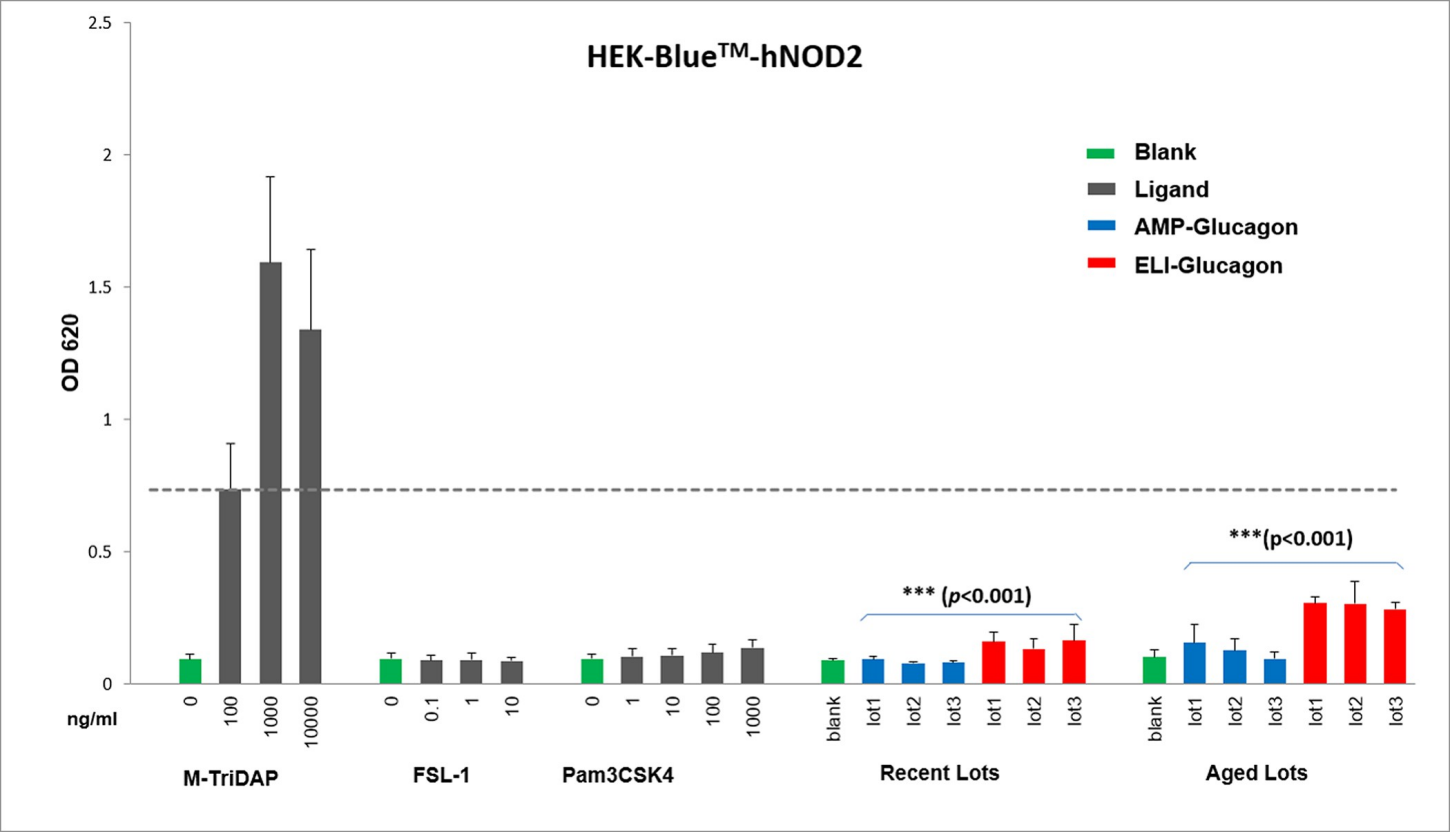
Comparison of innate immune response of AMP-Glucagon and ELI-Glucagon in HEK-Blue™-hNOD2 cells. Each bar represents mean ± stdev of 3–5 times of independent experiments. *p-*values by two-way ANOVA test for comparison between AMP-Glucagon and ELI-Glucagon lots.

As for the assessment of potential NLR related innate immunogenicity in glucagon products, the study results showed that all AMP-glucagon and ELI-glucagon product lots induced lower responses than that of the LLOD positive control (Table 4, Figs 2 and 3). Further in HEK-Blue™-hNOD1, although the one-way ANOVA test showed significant difference responses (*p* = 0.002) among the seven different treatments (including all six new lots of glucagon and the “medium only” negative control), there were no significant differences in response levels between the AMP-glucagon and ELI-glucagon new lots (two factor ANOVA, *p* = 0.74). For the aged glucagon lots treatment, both one-way ANOVA (*p* = 0.37) or two-way ANOVA (*p* = 0.58) test showed no significant difference among the treatment groups.

While there are no significant differences in the responses between AMP-glucagon and ELI-glucagon (either new or aged lots) in HEK-Blue™-hNOD1 cells, ELI-glucagon lots seemed to induce a notably higher innate immune response in HEK-Blue™-hNOD2 cells with *p*-values <0.001 based on both one-way and two-way ANOVA statistical test (as shown in Table 4 and Fig 3). In the new lots, AMP-glucagon had response levels of 0.080–0.095 compared to ELI-glucagon’s response levels of 0.133–0.165. In the aged lots, the response levels in AMP-glucagon (0.096–0.157) were again lower than the ELI-glucagon (0.284–0.307).

### Innate immunogenicity masking assessment assay

While the reporter cell lines are sensitive to IIRMIs, there is a possibility that the formulation of the drug product could mask the presence of impurities that may stimulate the innate immune system [6]. AMP-glucagon was used to investigate the possibility of masking in glucagon for the following reasons: (i) a primary purpose of the study is to investigate whether generic glucagon product induces wider or stronger innate immune response than that of the brand-name glucagon product; and (ii) the immune responses induced by the AMP-glucagon are similar or lower than ELI-glucagon such as in the HEK-Blue™-NOD2, which suggests that the masking would be more apparent in AMP-glucagon. The sensitivity of the three cell lines to their respective positive control ligands was tested in the presence of increasing concentrations of ligand alone or together with glucagon in four solution scenarios as listed in Table 5. In theory if masking were to occur, the response levels of the positive control with glucagon groups would appear significantly lower than the positive control alone.

**Table 5:** Solution types used for immunogenicity mask assessment assay.

Solution A	Positive control (PC) only
Solution B	PC together with AMP-glucagon API (active pharmaceutical ingredient)
Solution C	PC together with AMP-glucagon drug product;
Solution D	PC together with Placebo (AMP-glucagon formulation without API)

**Fig 4:**
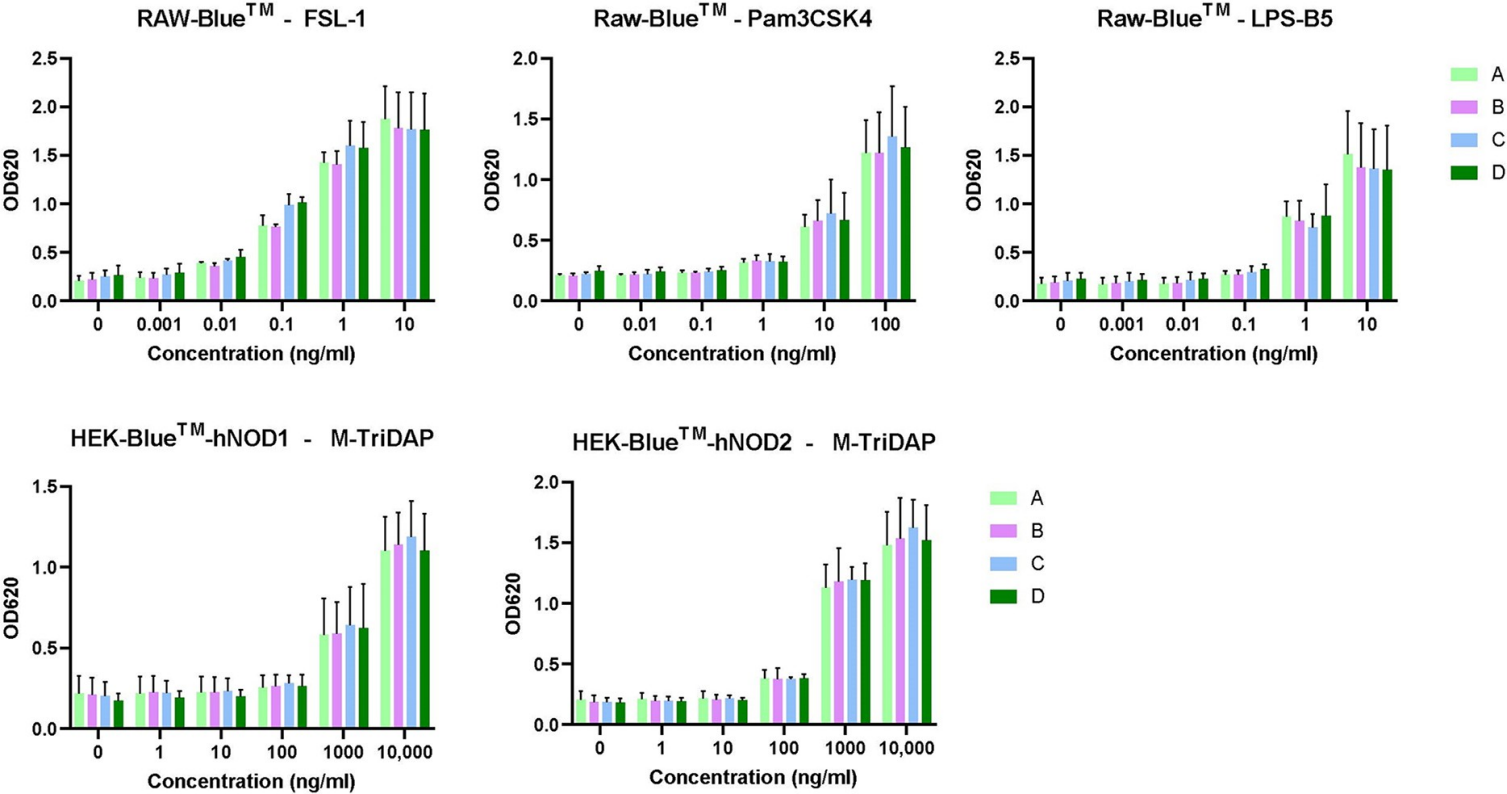
Innate immunogenicity masking assessment assay. Each bar represents mean ± stdev of 2–3 times of independent experiments.

The assay sensitivity to the four related positive controls (FSL1, Pam3CK4, LPS-B5 and M-TriDAP) in their respective cell lines remain at similar levels among all four different study solution scenarios (S1 Table and Fig 4). The responses of the cell lines were dose dependent and unchanged by the presence of AMP-glucagon. The addition of AMP-glucagon did not impact the activation of NF-κB, with the LLOD for FSL-1, Pam3CSK4, LPS-B5 in RAW-Blue™ remained at 0.001 ng/mL, 1 ng/mL, 1 ng/mL, respectively, and the LLOD for M-TriDAP in HEK-Blue™-hNOD1 and–hNOD2 remained at 1000 ng/mL and 100 ng/mL, respectively (S1 Table). The results indicate that the sensitivity of the three cell lines to detect IIRMIs are not masked or interfered by glucagon API or buffer formulation.

## Discussion and conclusion

This study is the first to use the cell-based assay to detect and assess the innate immunogenicity risk of IIRMIs in a chemically synthesized generic glucagon product. The results demonstrated that the synthetic glucagon product (new and aged lots) induced non-significant levels of innate immune responses when compared to the brand-name recombinant glucagon product, as well as the no-treatment negative control and/or the LLOD of the positive controls in all cell lines.

While different manufacturing processes can result in different levels of IIRMIs in each product, the synthetic glucagon (AMP-glucagon) and recombinant glucagon (ELI-glucagon) products induced comparable levels of innate immune responses in RAW-Blue™ and HEK-Blue™-hNOD1 cells. However, the recombinant glucagon products seemed to induce a notably higher innate immune responses in HEK-Blue™-hNOD2 compared to the synthetic glucagon. This is possible due to the fact that the ELI-glucagon is a recombinant peptide, which is produced via a complex path that often involves genetically modified host cell and complex growth/fermentation media [6,7]. While the downstream purification steps are designed to remove most impurities, it may not be sufficient to completely eliminate certain impurities from the recombinant product [6,7]. The synthetic glucagon, on the other hand, is produced through chemical synthesis, therefore does not have risk of host cell derived IIRMI contamination. It is worth noting that although the recombinant glucagon lots induced a higher innate immune response in HEK-Blue™-hNOD2 cells, the response levels were still way below the LLOD response level of the positive control ligand M-TriDAP, which is a strong NOD-like receptor (NLR) inducer.

In general, the cell-based assay system recommends the combined use of multiple cell lines to detect the presence of a broad range of IIRMIs, such as RAW-Blue^™^, THP-1 and MM6 cells [6]. For this study, we employed three cell lines (RAW-Blue™, HEK-Blue™-hNOD1 and HEK-Blue™-hNOD2) based on the chemical structure of the identified impurities presented in the generic synthetic glucagon product, which were short peptides such as lipoprotein or peptidoglycan. Other wide-spectrum PRR cell lines, such as THP-1 and MM6, which primarily sense non-peptide PAMPs were not relevant to this study. All three cell lines carry a SEAP reporter construct inducible by NF-κB. Stimulation of the cell lines with respective agonists will lead to the activation of the NF- κB, resulting in the SEAP secretion into the cell culture supernatant. The results in this study are consistent with the previous findings such that RAW-Blue™ is not sensitive on NOD1 and NOD2 signaling stimulation [6], and alternative cell lines such as HEK-Blue™-hNOD1 and HEK-Blue™-hNOD2 are needed to investigate the possibility of NLR related IIRMIs.

Bio-pharmaceutical products, whether proprietary or generic, have unique formulations and/or manufacturing processes. Therefore, cell lines selection for IIRMI risk evaluation should be flexible and based on potentially-presented or known impurities in the product, as well as the formulation buffer and API itself. This requires a thorough research and knowledge on the characteristics of the product to determine the most suitable experimental conditions to be used for the cell based assay.

Together, the data we obtained in this study indicates that the cell-based assay method could be used to screen chemically synthesized generic glucagon products for the presence of IIRMIs and assess the risk of immunogenicity.

## Supporting information

S1 Table Summary of innate immunogenicity masking assessment assay. The highlighted data indicate the potential LLOD concentrations of the ligand which elicited higher innate immune response with a *p*-value close to 0.05, when compared to its correspondent "0 ng/ml" negative control.
(PDF)
