# Supplementary material for: Assessment of innate immune response modulating impurities in glucagon for injection
Source: PLoS One. 2022 Nov 21;17(11):e0277922. doi: 10.1371/journal.pone.0277922 (PMC9678265; doi:10.1371/journal.pone.0277922)
Supplement: S1 Table — The highlighted data indicate the potential LLOD concentrations of the ligand which elicited higher innate immune response with a p-value close to 0.05, when compared to its correspondent "0 ng/ml" negative control. (PDF) [file pone.0277922.s001.pdf]

S1 Table - Summary of innate immunogenicity masking assessment assay

Cell lines / Positive controls (fomulation groups)		Dose ng/mL	fomulation A (PC alone)						fomulation B (PC in AMP-Glucagon API)						fomulation C (PC in AMP-Glucagon Drug Product)						fomulation D (PC in Placebo)					
			exp#1	exp#2	exp#3	**Ave.**	S.D.	p-value	exp#1	exp#2	exp#3	**Ave.**	S.D.	p-value	exp#1	exp#2	exp#3	**Ave.**	S.D.	p-value	exp#1	exp#2	exp#3	**Ave.**	S.D.	p-value
**Raw-Blue cells**	**PC1 (FSL-1),** synthetic diacylated peptide TLR2/6 ligand	0	0.253	0.221	0.159	**0.211**	0.048	(-)	0.284	0.230	0.155	**0.223**	0.065	(-)	0.300	0.277	0.185	**0.254**	0.061	(-)	0.361	0.272	0.165	**0.266**	0.098	(-)
		0.001	0.293	0.248	0.184	**0.242**	0.055	0.507	0.295	0.231	0.183	**0.236**	0.056	0.804	0.326	0.282	0.209	**0.272**	0.059	0.730	0.389	0.283	0.201	**0.291**	0.094	0.765
		0.01	0.406	0.384	0.379	**0.390**	0.014	0.003	0.395	0.349	0.333	**0.359**	0.032	0.032	0.432	0.418	0.402	**0.417**	0.015	0.011	0.525	0.457	0.384	**0.455**	0.071	0.053
		0.1	0.663	0.819	0.854	**0.779**	0.101	0.001	0.730	0.781	0.776	**0.762**	0.028	0.000	0.859	1.075	1.027	**0.987**	0.113	0.001	0.964	1.080	0.992	**1.012**	0.061	0.000
		1	1.387	1.548	1.340	**1.425**	0.109	0.000	1.471	1.504	1.246	**1.407**	0.140	0.000	1.595	1.858	1.348	**1.600**	0.255	0.001	1.648	1.799	1.278	**1.575**	0.268	0.001
		10	2.178	1.933	1.519	**1.877**	0.333	0.001	2.141	1.797	1.400	**1.779**	0.370	0.002	2.064	1.907	1.343	**1.771**	0.379	0.002	2.066	1.878	1.341	**1.762**	0.377	0.003
	**PC2 (Pam3CSK4)** synthetic triacylated peptide (TLR2/1 ligand)	0	0.220	0.213	0.198	**0.210**	0.011	(-)	0.229	0.202	0.197	**0.209**	0.017	(-)	0.212	0.238	0.221	**0.224**	0.014	(-)	0.265	0.275	0.198	**0.246**	0.042	(-)
		0.01	0.210	0.223	0.198	**0.210**	0.012	1.000	0.229	0.226	0.193	**0.216**	0.020	0.693	0.206	0.261	0.208	**0.225**	0.031	0.953	0.246	0.276	0.212	**0.245**	0.032	0.970
		0.1	0.235	0.250	0.219	**0.235**	0.015	0.090	0.233	0.242	0.216	**0.231**	0.013	0.162	0.231	0.271	0.221	**0.241**	0.026	0.364	0.253	0.283	0.224	**0.253**	0.030	0.818
		1	0.297	0.354	0.308	**0.320**	0.030	0.004	0.327	0.378	0.290	**0.332**	0.044	0.011	0.287	0.396	0.298	**0.327**	0.060	0.044	0.303	0.375	0.291	**0.323**	0.046	0.099
		10	0.600	0.717	0.515	**0.611**	0.102	0.002	0.646	0.835	0.498	**0.660**	0.169	0.010	0.576	1.041	0.542	**0.720**	0.279	0.037	0.560	0.926	0.518	**0.668**	0.225	0.033
		100	(-)	1.413	1.032	**1.223**	0.269	0.006	(-)	1.458	0.985	**1.222**	0.334	0.011	(-)	1.648	1.067	**1.358**	0.411	0.014	(-)	1.500	1.031	**1.266**	0.332	0.011
	**PC3 (LPS-B5),** Endotoxin, TLR4 ligand	0	0.103	0.225	0.199	**0.176**	0.064	(-)	0.116	0.238	0.211	**0.188**	0.064	(-)	0.121	0.269	0.239	**0.210**	0.078	(-)	0.170	0.293	0.220	**0.228**	0.062	(-)
		0.001	0.097	0.223	0.197	**0.172**	0.066	0.952	0.113	0.237	0.207	**0.186**	0.065	0.964	0.107	0.271	0.239	**0.206**	0.087	0.954	0.148	0.274	0.217	**0.213**	0.063	0.790
		0.01	0.106	0.227	0.200	**0.178**	0.064	0.975	0.120	0.231	0.210	**0.187**	0.059	0.985	0.121	0.279	0.248	**0.216**	0.084	0.931	0.171	0.281	0.228	**0.227**	0.055	0.984
		0.1	0.237	0.315	0.267	**0.273**	0.039	0.089	0.233	0.311	0.277	**0.274**	0.039	0.120	0.221	0.335	0.329	**0.295**	0.064	0.218	0.349	0.361	0.267	**0.326**	0.051	0.102
		1	1.036	0.853	0.729	**0.873**	0.154	0.002	1.039	0.814	0.629	**0.827**	0.205	0.007	0.905	0.743	0.631	**0.760**	0.138	0.004	1.192	0.894	0.537	**0.874**	0.328	0.028
		10	2.018	1.311	1.207	**1.512**	0.441	0.007	1.877	1.268	0.978	**1.374**	0.459	0.011	1.738	1.417	0.934	**1.363**	0.405	0.008	1.766	1.425	0.866	**1.352**	0.455	0.013
**HEK-Blue-hNOD1**	**PC4 (M-TriDAP)** Peptidoglycan NOD1/NOD2 ligand	0	0.344	0.161	0.152	**0.219**	0.108	(-)	0.331	0.159	0.150	**0.213**	0.102	(-)	0.305	0.147	0.159	**0.204**	0.088	(-)	0.225	0.149	0.153	**0.176**	0.043	(-)
		1	0.338	0.152	0.165	**0.218**	0.104	0.995	0.341	0.170	0.173	**0.228**	0.098	0.865	0.309	0.173	0.186	**0.223**	0.075	0.793	0.240	0.160	0.174	**0.191**	0.042	0.680
		10	0.337	0.159	0.190	**0.229**	0.095	0.911	0.332	0.173	0.180	**0.228**	0.090	0.855	0.322	0.181	0.195	**0.233**	0.078	0.691	0.248	0.175	0.175	**0.200**	0.042	0.535
		100	0.340	0.240	0.196	**0.259**	0.074	0.629	0.337	0.260	0.194	**0.264**	0.072	0.521	0.327	0.292	0.233	**0.284**	0.048	0.238	0.287	0.321	0.190	**0.266**	0.068	0.125
		1000	0.440	0.843	0.468	**0.583**	0.225	0.065	0.459	0.813	0.491	**0.588**	0.196	0.043	0.430	0.895	0.599	**0.642**	0.235	0.039	0.398	0.930	0.536	**0.621**	0.276	0.051
		10,000	1.018	1.341	0.945	**1.101**	0.211	0.003	1.091	1.358	0.978	**1.142**	0.195	0.002	1.091	1.442	1.027	**1.187**	0.224	0.002	1.005	1.365	0.935	**1.101**	0.231	0.002
**HEK-Blue-hNOD2**	**PC4 (M-TriDAP)** Peptidoglycan NOD1/NOD2 ligand	0	0.275	0.141	0.208	**0.208**	0.067	(-)	0.250	0.142	0.167	**0.186**	0.056	(-)	0.228	0.157	0.178	**0.188**	0.037	(-)	0.221	0.158	0.175	**0.185**	0.033	(-)
		1	0.264	0.160	0.212	**0.212**	0.052	0.937	0.245	0.167	0.178	**0.197**	0.042	0.812	0.235	0.176	0.188	**0.199**	0.031	0.697	0.226	0.170	0.177	**0.191**	0.031	0.834
		10	0.286	0.174	0.198	**0.219**	0.059	0.834	0.254	0.179	0.184	**0.206**	0.042	0.661	0.247	0.203	0.202	**0.217**	0.026	0.317	0.226	0.193	0.191	**0.203**	0.020	0.448
		100	0.459	0.350	0.341	**0.383**	0.066	0.032	0.463	0.380	0.288	**0.377**	0.087	0.034	0.376	0.362	0.392	**0.377**	0.015	0.001	0.343	0.398	0.404	**0.382**	0.034	0.002
		1000	1.330	1.116	0.948	**1.131**	0.191	0.001	1.468	1.158	0.922	**1.183**	0.274	0.004	1.299	1.199	1.094	**1.197**	0.103	0.000	1.262	1.282	1.031	**1.192**	0.139	0.000
		10,000	1.732	1.523	1.194	**1.483**	0.271	0.001	1.846	1.575	1.177	**1.533**	0.337	0.002	1.817	1.684	1.368	**1.623**	0.231	0.000	1.699	1.677	1.192	**1.523**	0.286	0.001
